# On Cluster Structures of Finnish Cancer Incidence Data

**DOI:** 10.1177/10732748261419587

**Published:** 2026-03-07

**Authors:** Tommi Huhtinen, Milla Laurikkala, Sirpa Heinävaara, Teemu J. Murtola, Pauliina Ilmonen

**Affiliations:** 1 Department of Mathematics and Systems Analysis, School of Science, Aalto University, Espoo, Finland; 2 Finnish Cancer Registry, Helsinki, Finland; 3 Prostate Cancer Research Center, Tampere University, Tampere, Finland

**Keywords:** agglomerative hierarchical clustering, cancer incidence, incidence pattern, risk factor, western lifestyle

## Abstract

**Introduction:**

The global burden of cancer is increasing. Part of this development is attributable to the estimated growth and aging of the population. In particular, aging is 1 of the main risk factors for cancer. However, there are many other risk factors beyond aging, including certain lifestyle and environmental factors. In addition, changes in diagnostic thresholds, increasing coverage of screening, and other similar factors affect cancer incidence rates. Therefore, even after excluding the effect of aging of the population, cancer incidence rates have not remained constant over time. To study these changes, the focus of this study is to identify and analyze cluster structures of the Finnish cancer incidence data from 1963 to 2023.

**Methods:**

To uncover the cluster structures, a proximity measure that is based on the shape of the curves is used. For unstandardized data, the proximity measure is shown to be invariant under simple location shift, and for standardized data, also under simple scaling, making the proximity measure suitable for assessing the similarities or dissimilarities of trends in time. As the group-building algorithm, agglomerative hierarchical clustering, combined with the average linkage method, is used.

**Results:**

The cluster structures were identified for 12 different subgroups, determined by age and sex. In many cases, cancers for which there exists a national screening program, including breast and cervical cancer, or an individualized testing tool, including prostate cancer, formed clusters of their own. Melanoma of the skin and lung & tracheal cancer are other 2 cancer types that often separated as their own clusters, possibly due to certain lifestyle factors.

**Conclusion:**

The study demonstrates the potential of the proposed proximity in the given context. In addition, the analysis of the cluster structures provides some insight into the Finnish cancer epidemiology.

## Plain Language Summary

Introduction The global burden of cancer is increasing. Main reasons behind this are that the number of people is growing, and a larger amount of people are old. Still, after excluding these effects, cancer incidence rates have changed over time. To study these changes, the focus of this study is to find hidden structures of the Finnish cancer incidence data from 1963 to 2023. Methods A statistical method called clustering is applied for finding the hidden structures of the Finnish cancer incidence data. Results The cluster structures were identified for 12 different subgroups, determined by age and sex. In many cases, cancers for which there exists a national screening program, including breast and cervical cancer, or an individualized testing tool, including prostate cancer, formed clusters of their own. Melanoma of the skin and lung & tracheal cancerare other two cancer types that often separated as their own clusters, possibly due to certain lifestyle factors.

## Introduction

The cancer burden is increasing worldwide. In 2022, 20 million new cancer cases were diagnosed and 9.7 million deaths were attributed to cancer.^1^ The authors estimate that by 2050, there will be an increase of 77%, corresponding to more than 35 million new cases, to the global cancer incidence figure of 2022. During the same time period, the world population is expected to grow from 8.0 billion to 9.7 billion and the share of people aged 65 years or older is expected to increase from 9.7% to 16.4%.^2^ As age is a significant risk factor,^3^ the growing number of people over 65 years of age explains a large part of the increasing incidence. In addition, many other, possibly modifiable, risk factors have been related to the increase in cancer burden. These include physical inactivity,^4^ an unbalanced diet and obesity,^5^ alcohol use,^6^ smoking,^7^ microplastics,^8^ and xenoestrogens.^9^

One motivation behind this study is that even after factoring in the aging of the population, cancer incidence rates have not remained constant over time. Instead, alterations in the carcinogenic exposure are expected due to changes that have occurred over the years in our lifestyle, the environment in which we live, or both. These alterations are reflected in the incidence rates. In addition, more frequent and widespread screening and testing, changes in the cancer diagnosis thresholds, and increased awareness of cancer and carcinogens are also aspects that affect the incidence rates.

In this study, cancer incidence time series of the most common cancers in Finland are studied. The objective is to identify and analyze cluster structures of the data. Analyzing cluster structures might shed some light on the underlying causes behind cancer incidence trends. Cluster analysis is commonly used in analyzing cancer incidence data, but most articles have only performed spatial or spatiotemporal clustering of incidence data (see for example^10-12^) instead of clustering longitudinal incidence rates. To our knowledge, there exists only 1 study^13^ where cluster analysis was performed on time series of cancer incidence. In this study, Finnish cancer incidence data from 1963 to 2023, obtained from the Finnish Cancer Registry,^14^ is exploited. To exclude the effect of the aging and possible changes in the age structure of the population, the incidence rates are studied in terms of age groups of 10 years, ranging from 30-39 to 70-79 years, and as rates per 100,000 person-years. It is expected that the development of the incidence rate over time for some cancers is similar to each other, but not across all cancers, meaning that distinguishable cluster structures would emerge. Furthermore, given age- and sex-related variations in hormonal activity, potential differences in the cluster structures between different subgroups, determined by age and sex, are investigated.

Within the scope of this study, answers are sought to the following 2 questions: (i) What kind of cluster structures can be identified from the Finnish cancer incidence data? (ii) What kind of differences, and similarities, can be observed in the cluster structures between different subgroups determined by age and sex? This study provides a wholly new analysis of the Finnish cancer incidence data by performing cluster analysis on the incidence rates over time, which has not been done before. Moreover, the clustering is performed to standardized data, allowing the patterns and shapes of the data to guide the clustering process without being dominated by quantity. In addition, a new tailored proximity measure, suitable for clustering cancer incidence time series, is proposed in this article.

## Methods

### Data Source

For the purposes of this study, cancer incidence data from the population-based Finnish Cancer Registry was used. This data is highly representable for the Finnish population since the coverage of registration is 96% for solid tumors.^15^ Data was extracted from the interactive cancer statistics application.^14^ The values are chosen to be categorized by sex, cancer site, year, and age group of 10 years, and are shown both as absolute number of cases and as rates per 100,000 person-years. The years covered are from 1963 to 2023. Then, after the extraction and before any further analyses, the raw data is preprocessed. A respective script is provided online in a GitHub repository as Supplemental material.^16^ In preprocessing, basal cell carcinomas of the skin and genitals, borderline tumor of the ovary, carcinoma in situ of the breast as well as non-invasive neoplasms of cervix uteri, vagina, and vulva are excluded from further analyses. These cancer types seldom spread to other parts of the body,^17^ tend to progress slowly from the low-grade precursor stage,^18^ or remain localized.^19^ All other cancer types are included into the analysis part of this study.

A relevant note regarding the extracted cancer incidence data is the following. In Finland, reporting all new diagnosed cancer cases to the national cancer registry, that is, the Finnish Cancer Registry, is mandatory by law. Therefore, the data used in this study represents complete population coverage rather than samples. Consequently, all fluctuations observed in the data reflect the absolute variation in the overall incidence. In addition, it should be noted that in the youngest age groups the overall incidence is low, and random annual variation can therefore outweigh any underlying trends.

### Preliminary Analysis

**Figure 1.**
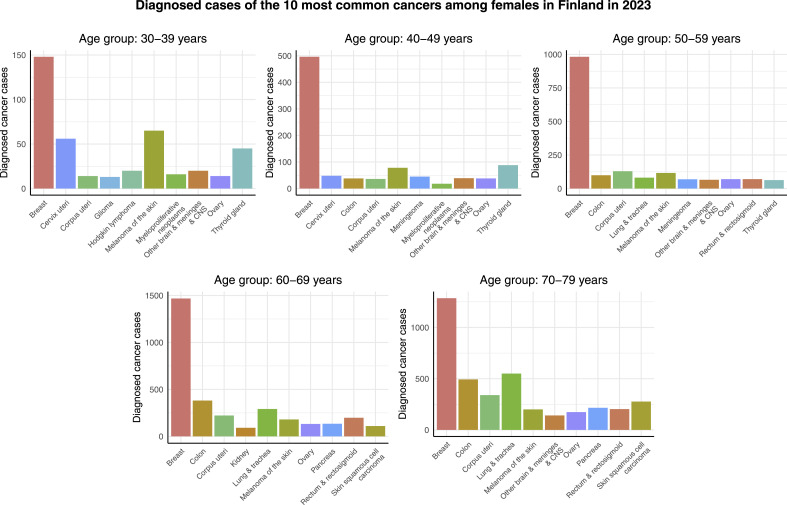
Cancer cases of the 10 most common cancers among females in different age groups in Finland in 2023

Figure 1 displays the cancer cases of the 10 most common cancers among females in different age groups in Finland in 2023. Here the label ’other brain & meninges & CNS’ refers to other and unspecified tumors of the brain, meninges and central nervous system, excluding glioma, meningeoma, and central nervous system and nerve sheath tumor, as these excluded cancers have their own categories. An initial observation from Figure 1 is that breast cancer is clearly the most common cancer in all age groups. It is the most dominating in the age group of 40-49 years where there are almost 500 breast cancer cases compared to fewer than 100 cases of the second most common cancer, and in the age group of 50-59 years with almost 1000 breast cancer cases compared to 125 cases of the second most common cancer. In the age groups of 60-69 and 70-79 years, there are more than 1000 cases of breast cancer and around 500 cases of the second most common cancer. In the youngest age group, 30-39 years, breast cancer is the least dominating: fewer than 150 cases compared to more than 50 cases of the second most common cancer. The ranks of other cancers vary between age groups. Melanoma of the skin is the second most common in the age group of 30-39 years and the third most common cancer in the age groups of 40-49 and 50-59 years. Cervical cancer is the second most common cancer in the age group of 30-39 years. In the 2 oldest age groups, colon and lung & tracheal cancer are more common than in the younger age groups: colon cancer ranks as the second and lung & tracheal cancer as the third most common cancer within 60-69-year-old females, and in the age group of 70-70 years, lung & tracheal cancer is the second most common while colon cancer is the third most common cancer. Lastly, it can be observed that, in general, the older the age group, the more there are cancer cases, emphasizing the role of aging in cancer development.

**Figure 2.**
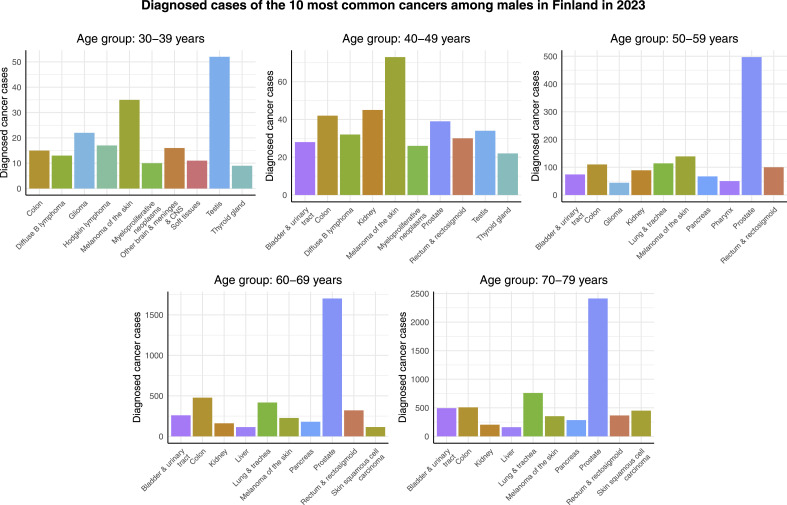
Cancer cases of the 10 most common cancers among males in different age groups in Finland in 2023

Figure 2 shows the cancer cases of the 10 most common cancers among males in different age groups in Finland in 2023. Contrary to females, there is no individual cancer type that would be the most common 1 across all of the age groups. Instead, testicular cancer, melanoma of the skin, and prostate cancer are the leading cancer types in terms of diagnosed cancer cases in the age groups of 30-39 years, 40-49 years, and the rest of the age groups, respectively. In addition, the most common cancers in the case of males are not as dominating as in females. The differences in number of cases between the most and second most common cancers range from a few dozens in the 2 youngest age groups, to 350 in the age group of 50-59 years, and to around 1000 in the 2 oldest age groups. On the other hand, also in the case of males, it can be observed that there are more cancer cases among older people. In addition, lung & tracheal cancer is relatively common not only among males aged 60-69 and 70-79 years, but also in the age group of 50-59 years.

The time series representing the incidence rates per 100,000 person-years of the 10 most common cancers among females and males in different age groups from 1963 to 2023 are provided online as Supplemental material.^16^ In the case of females, breast cancer is consistently the most common cancer throughout the observation period but the ranks of other cancers change. For example, in the younger age groups, cervical cancer is the second most common cancer in the 1960s, and melanoma of the skin becomes the second most common cancer towards the end of the interval, in the 2010s and 2020s. In the case of males, the ranks of cancers in the younger age groups vary a lot during the time interval. In the older age groups, the most common cancer until the 1990s is lung & tracheal cancer, which is then overtaken by prostate cancer.

### Agglomerative Hierarchical Clustering

Cluster analysis is a commonly used data analysis method, where the aim is to identify groups of similar observations, ie, clusters, from a given dataset.^20^ Clustering is considered to be an unsupervised machine learning method because instead of separating the data into pre-existing classes, the groups arise naturally from the data.^20^ This means that there are no given labels for the groups. In mathematical terms, the aim is to form clusters that minimize within-group homogeneity and maximize between-group heterogeneity. To achieve this, 2 essential steps are needed: the choice of an appropriate proximity measure, and the choice of an appropriate group-building algorithm. These choices should be guided by the context at hand. In general, the purpose of the proximity measure is to measure the distance between objects based on their (dis)similarities. The purpose of the group-building algorithm, on the other hand, is to assign objects into clusters in a way that allows for achieving the objective of the cluster analysis.

In this study, the objects to be assigned to clusters are time series representing the incidence rates per 100,000 person-years of the most common cancers. Therefore, an appropriate proximity measure is 1 that measures distance between the time series based on the overall shape of the curves, while excluding the effect of possible differences in the baseline rates. One such measure is:

(1) $$\begin{array}{ll} D\left(X,Y\right) & =\frac{1}{T_{K}-T_{0}}\int_{T_{0}}^{T_{K}}\left[d\left(X\left(t\right),Y\left(t\right)\right)\right.\\ & \;-\left.d\left(T\left(X\right),T\left(Y\right)\right)\right]\;dt, \end{array}$$

where *X*(*t*) and *Y*(*t*) are continuous functions over the time interval [*T*_0_, *T*_*K*_], *d*(⋅, ⋅) is some one-dimensional distance measure, and *T*(⋅) is some one-dimensional location measure. The discrete approximation of the proximity measure (1) can be given using the Riemann sum approximation of the integral^21^:

(2) $$\begin{array}{ll} D\left(X,Y\right) & =\frac{1}{T_{K}-T_{0}}\int_{T_{0}}^{T_{K}}\left[d\left(X\left(t\right),Y\left(t\right)\right)\right.\\ & \;-\left.d\left(T\left(X\right),T\left(Y\right)\right)\right]dt\\ & \approx \frac{1}{T_{K}-T_{0}}\overset{k}{\underset{i=1}{\sum }}\left[d\left(x_{i},y_{i}\right)\right.\\ & \;-\left.d\left(T\left(X\right),T\left(Y\right)\right)\right]\frac{T_{K}-T_{0}}{k}\\ & =\frac{1}{k}\overset{k}{\underset{i=1}{\sum }}\left[d\left(x_{i},y_{i}\right)-d\left(T\left(X\right),T\left(Y\right)\right)\right], \end{array}$$

where *X* = [*x*_1_, …, *x*_*k*_] and *Y* = [*y*_1_, …, *y*_*k*_] now represent collections of *k* equispaced yearly observations of incidence rates per 100,000 person-years.

Thus far, *d*(⋅, ⋅) and *T*(⋅) have been considered as some general distance and location measure, respectively. One possible choice is choosing $d\left(x_{i},y_{i}\right)={\left(x_{i}-y_{i}\right)}^{2},\forall i\in k$ , and *T*(*X*) = $\bar{x}$ , where $\bar{x}=\left(\sum_{i=1}^{k}x_{i}\right)/k$ is the mean of the *k* observations. Then, the proximity measure (2) becomes:

(3) $$D\left(X,Y\right)=\frac{1}{k}\overset{k}{\underset{i=1}{\sum }}\left[{\left(x_{i}-y_{i}\right)}^{2}-{\left(\bar{x}-\bar{y}\right)}^{2}\right].$$

The proximity measure (3) is used in the data analysis part of this study. It can be shown that the proximity measure (3) is zero when 2 curves are identical up to vertical shift by a constant *b* ≠ 0. That is, when *y*_*i*_ = *x*_*i*_ + *b*, *∀i* ∈ *k*, it follows that:

$$\begin{array}{ll} D\left(X,Y\right) & =\frac{1}{k}\overset{k}{\underset{i=1}{\sum }}\left[{\left(x_{i}-y_{i}\right)}^{2}-{\left(\bar{x}-\bar{y}\right)}^{2}\right]\\ & =\frac{1}{k}\overset{k}{\underset{i=1}{\sum }}\left[{\left(x_{i}-\left(x_{i}+b\right)\right)}^{2}-{\left(\bar{x}-\left(\bar{x}+b\right)\right)}^{2}\right]\\ & =\frac{1}{k}\overset{k}{\underset{i=1}{\sum }}\left(b^{2}-b^{2}\right)\\ & =0. \end{array}$$

Remark Alternative choices for the distance measure *d*(⋅, ⋅) and location measure *T*(⋅) would be, eg, *d*(*x*_*i*_, *y*_*i*_) = |*x*_*i*_ − *y*_*i*_|, *∀i* ∈ *k*, and *T*(*X*) = Med(*X*), where Med(*X*) is the median of the *k* observations. However, choosing $d\left(x_{i},y_{i}\right)={\left(x_{i}-y_{i}\right)}^{2}$ over *d*(*x*_*i*_, *y*_*i*_) = |*x*_*i*_ − *y*_*i*_| enhances the detection of large differences in curve shapes. Additionally, opting for $T\left(X\right)=\bar{x}$ rather than *T*(*X*) = Med(*X*) enables the incorporation of information along all points in the curve, while the median reflects only an individual (central) data point.

One may also consider standardizing the variables *x*_*i*_, *y*_*i*_, *∀i* ∈ *k* by setting:

(4) $${\hat{x}}_{i}=\frac{x_{i}-T\left(X\right)}{\sqrt{s\left(X\right)}},\forall i\in k,$$

where ${\hat{x}}_{i}$ is the standardized variable, *T*(*X*) is a one-dimensional location measure, and *s*(*X*) is a one-dimensional scatter measure. Possible choices are $T\left(X\right)=\bar{x}$ and $s\left(X\right)=\left(\sum_{i=1}^{k}{\left(x_{i}-\bar{x}\right)}^{2}\right)/k$ , that is, the sample mean and sample variance, respectively. Then, the standardization formula (4) becomes:

(5) $${\hat{x}}_{i}=\frac{x_{i}-\frac{1}{k}\sum_{i=1}^{k}x_{i}}{\sqrt{\frac{1}{k}\sum_{i=1}^{k}{\left(x_{i}-\bar{x}\right)}^{2}}},\forall i\in k.$$

The standardization formula (5) is used in the data analysis part of this study.

Remark An alternative choice for the scatter measure *s*(*X*) would be, eg, *s*(*X*) = MAD(*X*), where MAD(*X*) = Med(|*x*_*i*_ − Med(*X*)|, …, |*x*_*k*_ − Med(*X*)|) is the Median Absolute Deviation (MAD).^22^

In this study, the clustering is performed using both unstandardized and standardized data. In the case of standardized data, the proximity measure (3) is zero when 2 curves are identical, not only up to a vertical shift by a constant *b* ≠ 0 but also up to scaling by a constant *a* > 0. That is, when $y_{i}=ax_{i}+b,\forall i\in k,\hat{X}=\left[{\hat{x}}_{i},\ldots ,{\hat{x}}_{k}\right]$ , and $\hat{Y}=\left[{\hat{y}}_{1},\ldots ,{\hat{y}}_{k}\right]$ , where the variables ${\hat{x}}_{i}$ , ${\hat{y}}_{i},\forall i\in k$ , have been standardized according to the formula (5), it follows that:

$$\begin{array}{ll} D\left(\hat{X},\hat{Y}\right) & =\frac{1}{k}\overset{k}{\underset{i=1}{\sum }}\left[d\left({\hat{x}}_{i},{\hat{y}}_{i}\right)-d\left(T\left(\hat{X}\right),T\left(\hat{Y}\right)\right)\right]\\ & =\frac{1}{k}\overset{k}{\underset{i=1}{\sum }}\left[{\left({\hat{x}}_{i}-{\hat{y}}_{i}\right)}^{2}-{\left(\bar{\hat{x}}-\bar{\hat{y}}\right)}^{2}\right]\\ & =\frac{1}{k}\overset{k}{\underset{i=1}{\sum }}\left[{\left(\frac{x_{i}-\bar{x}}{\sqrt{s\left(X\right)}}-\left(\frac{ax_{i}+b-\left(a\bar{x}+b\right)}{\sqrt{s\left(Y\right)}}\right)\right)}^{2}\right.\\ & \left.-{\left(0-0\right)}^{2}\right]\\ & =\frac{1}{k}\overset{k}{\underset{i=1}{\sum }}{\left[\frac{x_{i}-\bar{x}}{\sqrt{s\left(X\right)}}-\left(\frac{a\left(x_{i}-\bar{x}\right)}{\sqrt{s\left(Y\right)}}\right)\right]}^{2}\\ & =\frac{1}{k}\overset{k}{\underset{i=1}{\sum }}{\left[\frac{x_{i}-\bar{x}}{\sqrt{s\left(X\right)}}-\left(\frac{a\left(x_{i}-\bar{x}\right)}{\sqrt{a^{2}s\left(X\right)}}\right)\right]}^{2}\\ & =\frac{1}{k}\overset{k}{\underset{i=1}{\sum }}{\left[\frac{x_{i}-\bar{x}}{\sqrt{s\left(X\right)}}-\left(\frac{a\left(x_{i}-\bar{x}\right)}{a\sqrt{s\left(X\right)}}\right)\right]}^{2}\\ & =\frac{1}{k}\overset{k}{\underset{i=1}{\sum }}{\left[\frac{x_{i}-\bar{x}}{\sqrt{s\left(X\right)}}-\left(\frac{\left(x_{i}-\bar{x}\right)}{\sqrt{s\left(X\right)}}\right)\right]}^{2}\\ & =0. \end{array}$$

The practical implication of this property is that for standardized data, the proposed proximity measure is invariant under simple location shift and scaling, making it suitable for assessing the similarities or dissimilarities of (possibly nonlinear) trends in time. That is, the clustering is based on the shape of the curves, rather than just allocating common cancers in 1 cluster and rare cancers in another cluster. However, it should be noted that this property is dependent on the choice of the squared distances and mean as the distance and location measure, respectively, and therefore does not generalize for all possible choices of the distance and location measures.

As discussed above, in addition to the proximity measure, an appropriate group-building algorithm is needed. In their article, Zhang and Parnell^23^ review different algorithms for clustering functional data (ie, data that can be viewed as a collection of observed (continuous) functions^24^), such as cancer incidence time series. Additionally, an experimental part is included in their article, in which it is observed that agglomerative hierarchical clustering, combined with a suitable linkage method, outperforms some other algorithms, including *k*-means, subspace clustering, and density-based clustering.^23^ The linkage method determines the criteria for merging clusters as the algorithm proceeds. This can be based on the minimum, average, or maximum distance between objects in different clusters (single, average, and complete linkage, respectively).^25^ Research suggests that the average linkage method, in which the distance between the clusters or individual objects *Q* and *R* is defined as:

(6) $$d_{A}\left(Q,R\right)=\frac{1}{\left|R\|Q\right|}\underset{X\in R,Y\in Q}{\sum }D\left(X,Y\right),$$

where |*R*| and |*Q*| are the number of objects in clusters *R* and *Q*, respectively, is a reasonable choice when 1 or 2 large clusters are anticipated and the data is non-periodic, whereas Ward’s method is preferred for periodical data.^25^

In light of the discussion above, and the fact that the use of agglomerative hierarchical clustering does not require knowing the number of clusters beforehand, agglomerative hierarchical clustering is chosen for the data analysis part of this study. As no periodicity is expected among the cancer incidence data, the algorithm is used with the average linkage method. Using this approach, the average distances *d*_*A*_(*Q*, *R*) (6) are computed between all cluster pairs, and the pair of the smallest *d*_*A*_(*Q*, *R*) is merged into 1. This process is repeated until the desired amount of clusters is obtained. The used clustering algorithm is provided online as Supplemental material.^16^

## Results

The results of the cluster analyses are shown using both unstandardized and standardized data. The standardization is performed as follows: Consider breast cancer incidence rate per 100,000 person-years among females aged 40-49 years in Finland. First, the sample mean and the sample standard deviation of the yearly breast cancer incidence rate per 100,000 person-years in this age group is calculated. Then each breast cancer incidence data point in this age group is standardized by subtracting the sample mean and dividing by the sample standard deviation. Standardization is done separately for each cancer in each age group. This standardization enables 1 to assess the similarities or dissimilarities in the shape (trend) of the time series curves such that the commonness of the cancer does not affect the analyses. On the other hand, for unstandardized data, the commonness of the cancer does affect the clustering results. For each age group, the cancer incidence data were divided into 2, 3, and 4 clusters.

**Figure 3.**
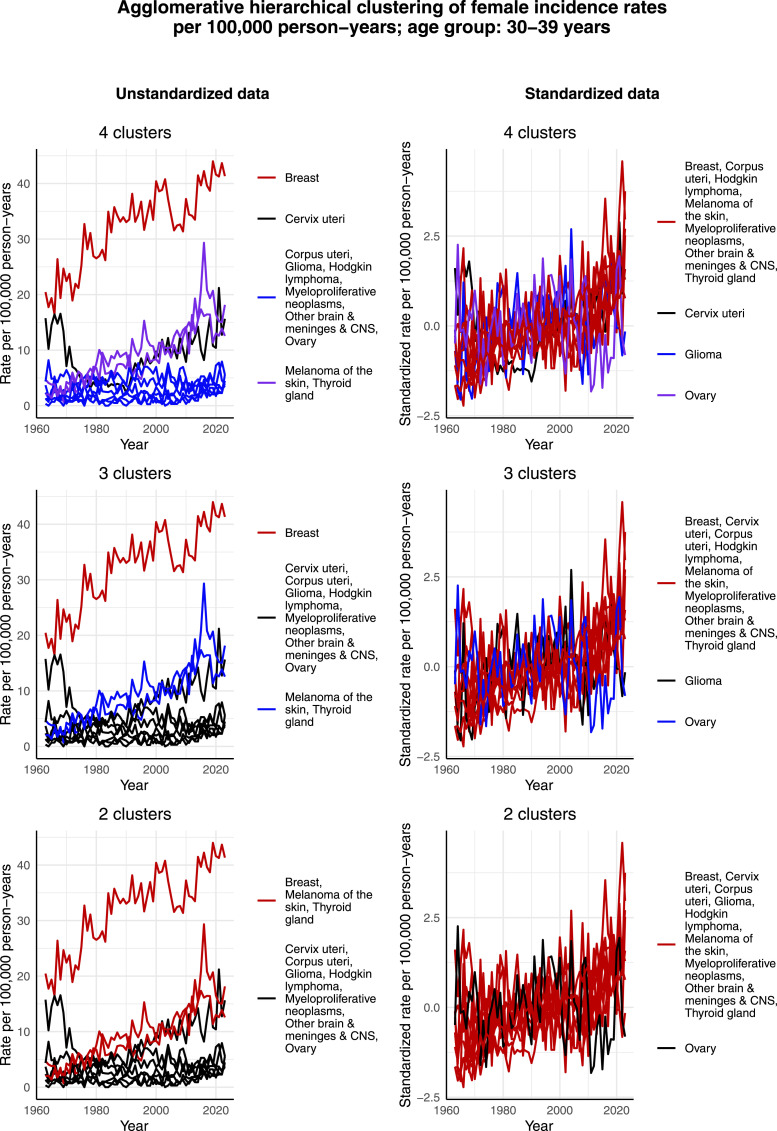
Agglomerative hierarchical clustering applied to the incidence rates per 100,000 person-years of the most common cancers among females aged 30-39 years in Finland

Figure 3 visualizes the clustering results for females aged 30-39 years. In the case of the unstandardized data, the cluster structure containing 4 clusters appears to be particularly reasonable. That is, there is 1 cancer, namely breast cancer, whose incidence rate per 100,000 person-years is clearly higher than that of the other most common cancers. Second, the incidence rate per 100,000 person-years of melanoma of the skin and thyroid gland cancer has increased in a similar way as that of breast cancer over time, but the scale is smaller. Moreover, when data is standardized, these cancers appear in the same cluster, see Figure 3. Third, contrary to the other most common cancers, the incidence rate per 100,000 person-years of cervical cancer appears to have decreased during the 1960s and 1970s with a 78% decrease from 1963 to 1980, and then increased from the 1990s onward with a 547% increase during the period from 1990 to 2023. Lastly, the incidence rate per 100,000 person-years of the rest of the most common cancers is relatively low, and appears also relatively constant over the studied time interval. However, based on the standardized data, it can be observed that most standardized incidence rates per 100,000 person-years of the most common cancers in this age group exhibit a slightly increasing trend over time. The only exceptions are cervical cancer, for the reasons discussed above, as well as glioma and ovarian cancer, for which the rates appear to have remained constant.

**Figure 4.**
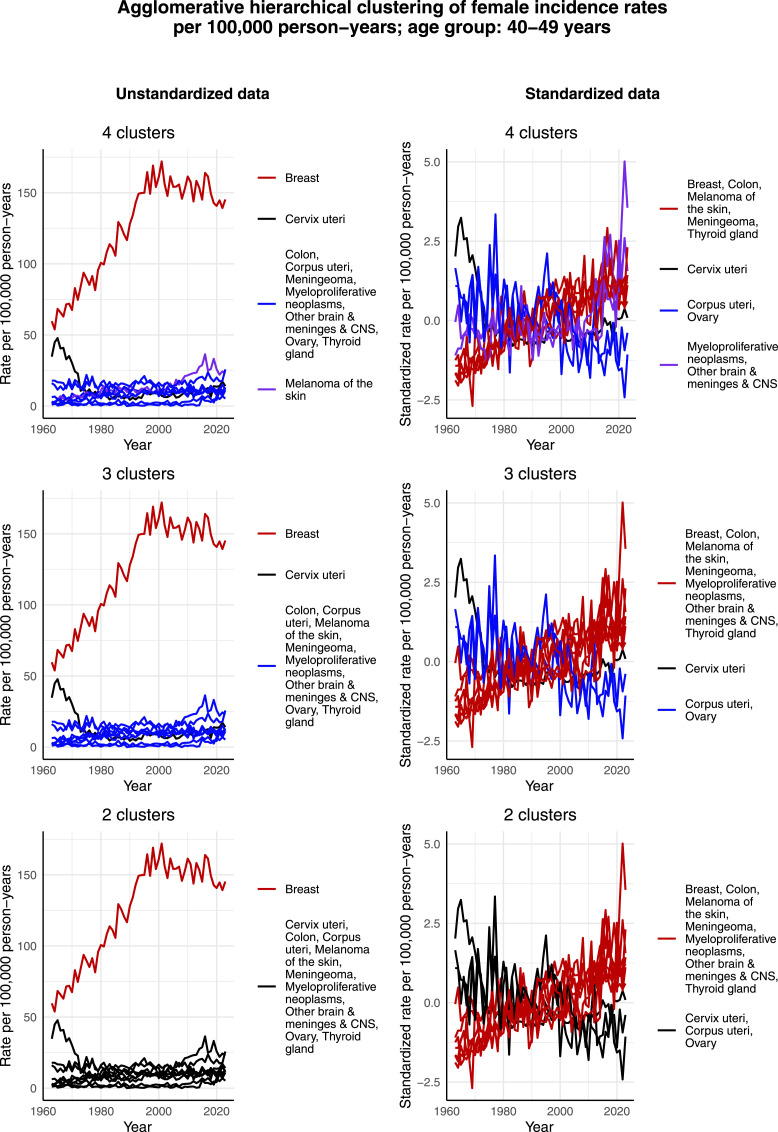
Agglomerative hierarchical clustering applied to the incidence rates per 100,000 person-years of the most common cancers among females aged 40-49 years in Finland

Figure 4 illustrates the clustering results for females aged 40-49 years. The incidence rate per 100,000 person-years of breast cancer, melanoma of the skin, and cervical cancer exhibit similar trends as in the age group of 30-39 years, and thus separate as their own clusters in the case of 4 clusters and unstandardized data. Moreover, breast cancer has a much larger incidence rate per 100,000 person-years than the other most common cancers, causing the scale to guide the clustering process in the case of unstandardized data so that breast cancer separates as its own cluster in the case of 4, 3 and 2 clusters. The effect of scale becomes more obvious when compared to the clustering of standardized data. There, the scales are normalized, and the standardized incidence rate of breast cancer no longer stands out. Indeed, then breast cancer is assigned to the same cluster as other cancers, whose standardized incidence rate per 100,000 person-years demonstrates an increasing trend over time. This highlights the fact that the commonness of the cancer, ie, the scale, affects the clustering results in the case of unstandardized data. In terms of the standardized data, the algorithm is able to capture the decreasing trend of the standardized incidence rate per 100,000 person-years of corpus uteri and ovarian cancer over time, and thereby assigns these 2 cancers to the same cluster. It also seems that since the standardized incidence rate per 100,000 person-years of cervical cancer is not decreasing during the whole time interval, especially during the 2000s, it is assigned to the same cluster as corpus uteri and ovarian cancer only when 2 clusters are identified.

**Figure 5.**
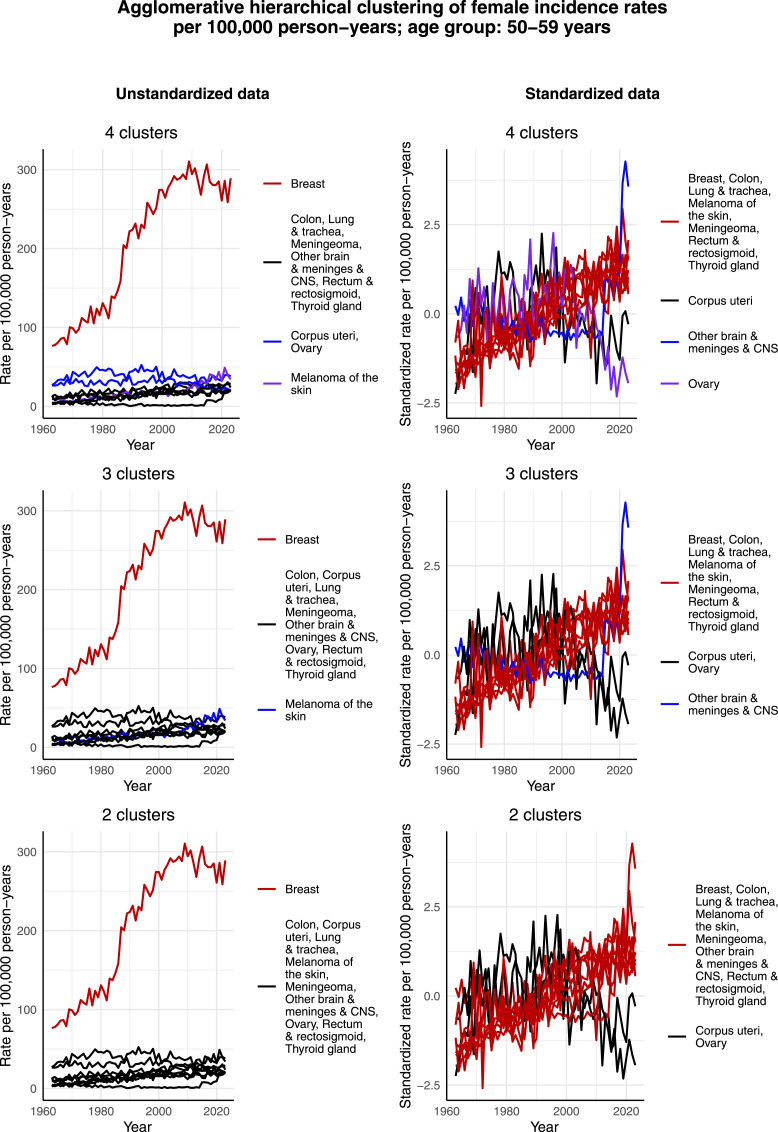
Agglomerative hierarchical clustering applied to the incidence rates per 100,000 person-years of the most common cancers among females aged 50-59 years in Finland

Figure 5 presents the clustering results for females aged 50-59 years. Again, breast cancer forms its own cluster in the case of the unstandardized data in all 3 cases of 2, 3, and 4 clusters. Moreover, in relative terms, the incidence rate per 100,000 person-years of melanoma of the skin appears to have increased more over time than that of some other cancers, thus separating as its own cluster in the cases of 3 and 4 clusters. In terms of the standardized data, corpus uteri and ovarian cancer form separate clusters in the case of 4 clusters, but a joint cluster when 2 or 3 clusters are identified. This could result from the fact that both cancers have a decreasing standardized incidence rate per 100,000 person-years especially during the 2000s.

**Figure 6.**
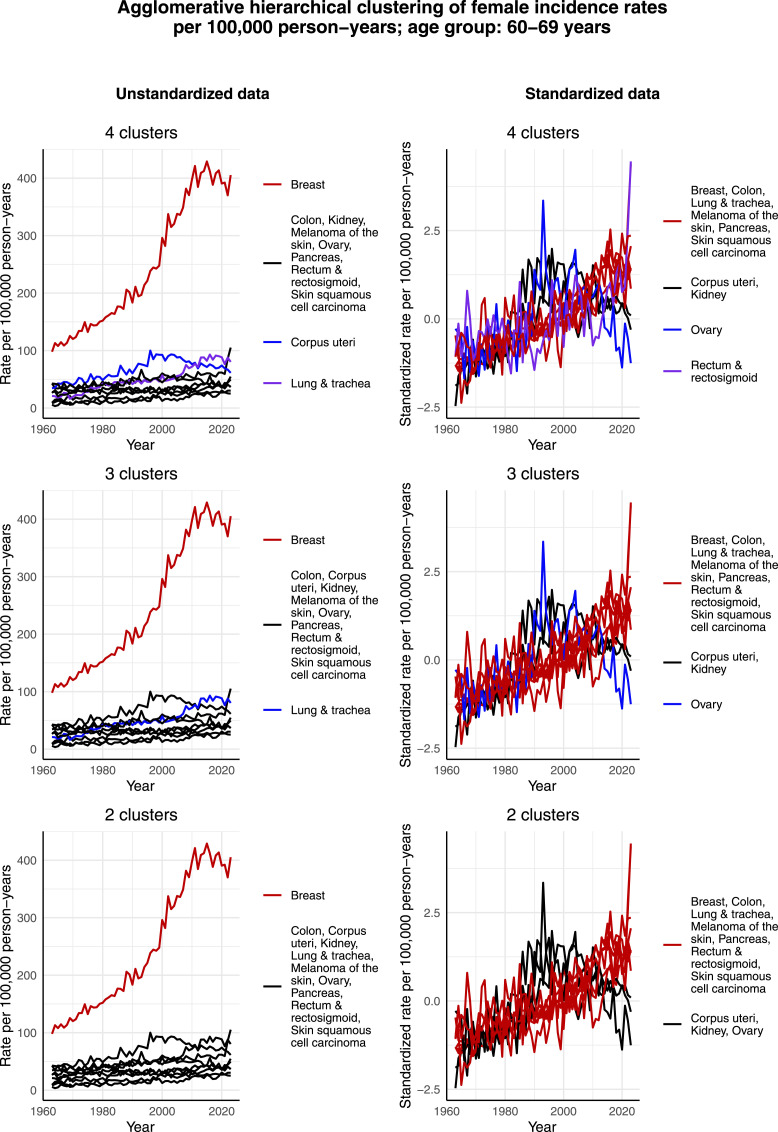
Agglomerative hierarchical clustering applied to the incidence rates per 100,000 person-years of the most common cancers among females aged 60-69 years in Finland

Figure 6 displays the clustering results for females aged 60-69 years. In addition to breast cancer forming its own cluster, and contrary to the age groups from 30-39 to 50-59 years, lung & tracheal cancer also shows a tendency to form its own cluster in the cases of 3 and 4 clusters when unstandardized data is considered. The reason seems to be the increase in its incidence rate per 100,000 person-years, which appears to be in relative terms larger than that of the other most common cancers. However, when it comes to the standardized data, lung & tracheal cancer no longer separate as its own cluster. Rather, it is again corpus uteri and ovarian cancer, as well as kidney cancer, that deviate from the rest of the most common cancers. This can be seen from the fact that the standardized incidence rate of these 3 cancers per 100,000 person-years has increased only during the first half of the time interval and then decreased during the second half, whereas the standardized incidence rate per 100,000 person-years of the rest of the most common cancers has increased through the whole time interval. Consequently, corpus uteri, kidney and ovarian cancer form either 2 clusters or 1 cluster while the majority of the other most common cancers form 1 big cluster together.

**Figure 7.**
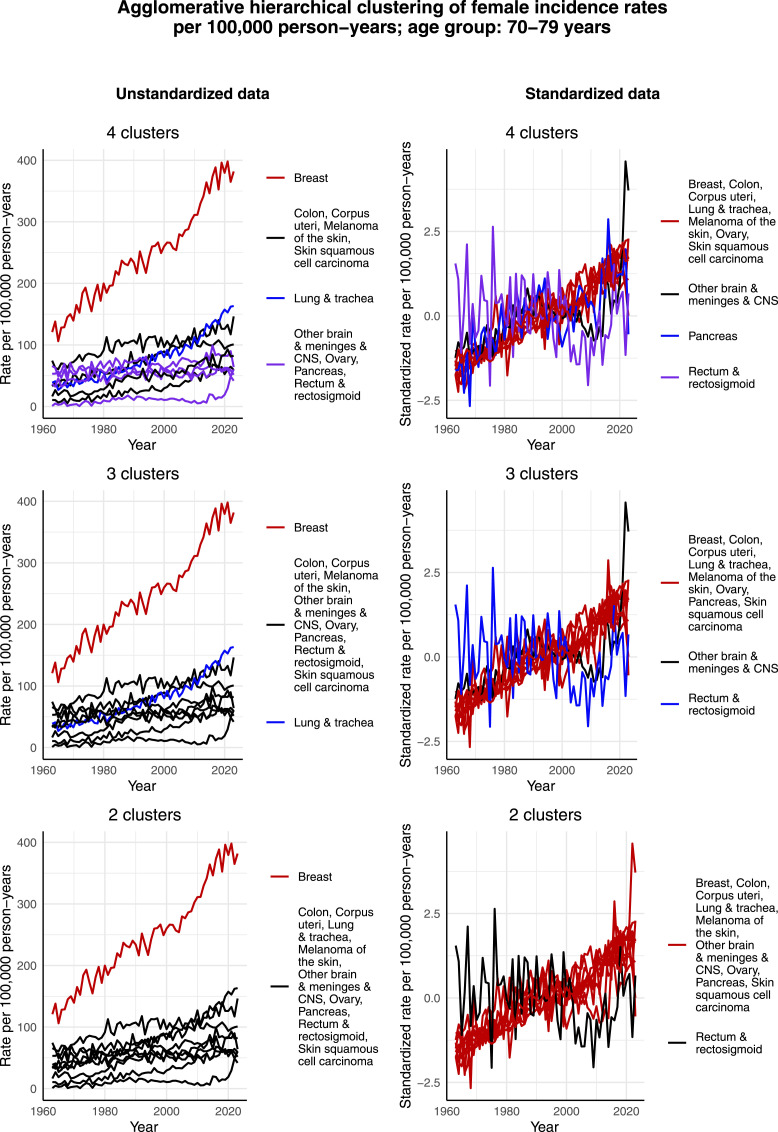
Agglomerative hierarchical clustering applied to the incidence rates per 100,000 person-years of the most common cancers among females aged 70-79 years in Finland

Figure 7 shows the clustering results for females aged 70-79 years. Compared to the other groups, it is interesting that in this age group there seem to be many cancers, whose incidence rate per 100,000 person-years has increased in a way that is observable. Consequently, in the case of unstandardized data and 4 clusters, there are not simply 3 clusters, each formed by an individual cancer type, and then the rest of the cancers assigned to a joint cluster, but in fact 2 clusters that are formed by multiple cancer types. Colon and corpus uteri cancer, melanoma of the skin and skin squamous cell carcinoma form 1 cluster, and other brain & meninges & CNS, ovarian, pancreatic and rectal & rectosigmoid cancer form another cluster. The 2 single-type clusters are formed by breast cancer and lung & tracheal cancer. Another interesting observation concerns standardized data and rectal & rectosigmoid cancer. Namely, not only does rectal & rectosigmoid cancer form its own cluster given its slightly decreasing standardized incidence rate per 100,000 person-years over time, but this development is also somewhat opposite as in the age group of 60-69 years, as illustrated by Figure 6.

**Figure 8.**
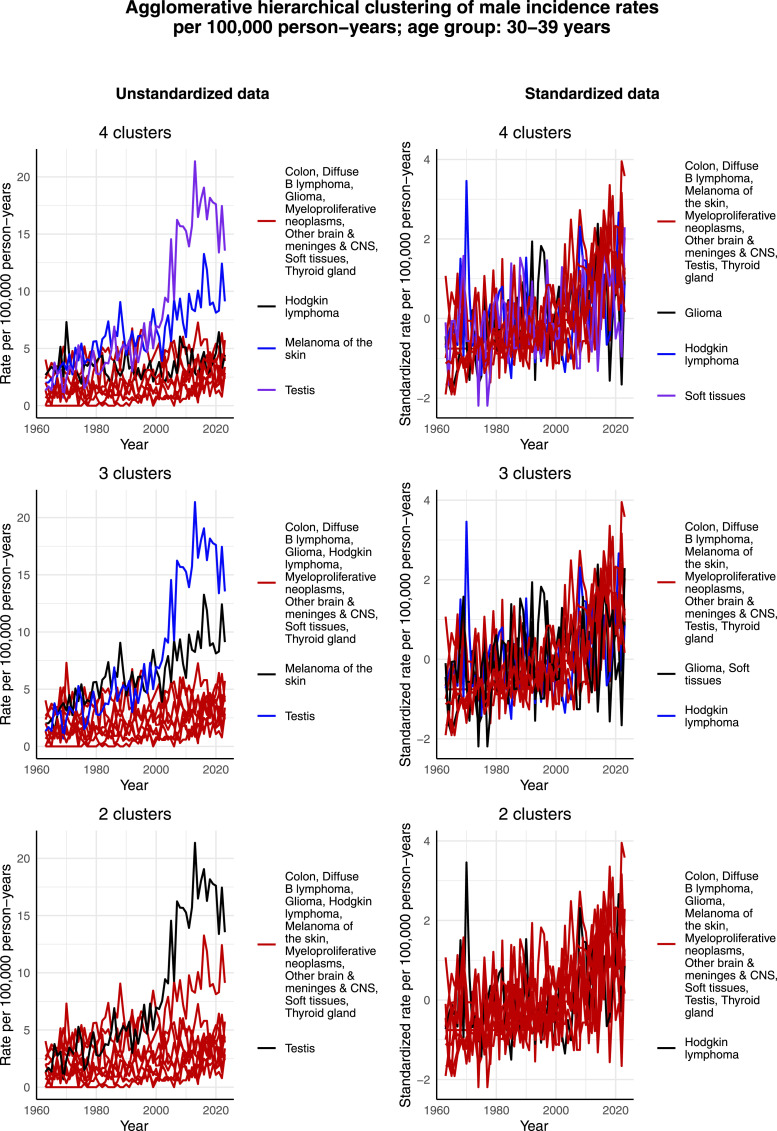
Agglomerative hierarchical clustering applied to the incidence rates per 100,000 person-years of the most common cancers among males aged 30-39 years in Finland

Figure 8 presents the clustering results for males aged 30-39 years. It appears that testicular cancer forms its own cluster in the case of the unstandardized data due to its highest incidence rate per 100,000 person-years during the 2000s. There are 2 other cancers that form their own clusters in the case of 4 clusters: melanoma of the skin and Hodgkin lymphoma, with the former also forming its own cluster in the case of 3 clusters. However, when looking at the standardized incidence rates per 100,000 person-years, the data look noisy and there are no clear or differing trends visible. Taking into account the very small incidence of this young age group, it seems that random fluctuations guide the clustering process instead of actual trends in the data. This becomes even more evident when comparing the incidence rates of this age group with those of older age groups, see for example Figures 11 and 12, where different trends that affect the clustering process are clearly visible.

**Figure 9.**
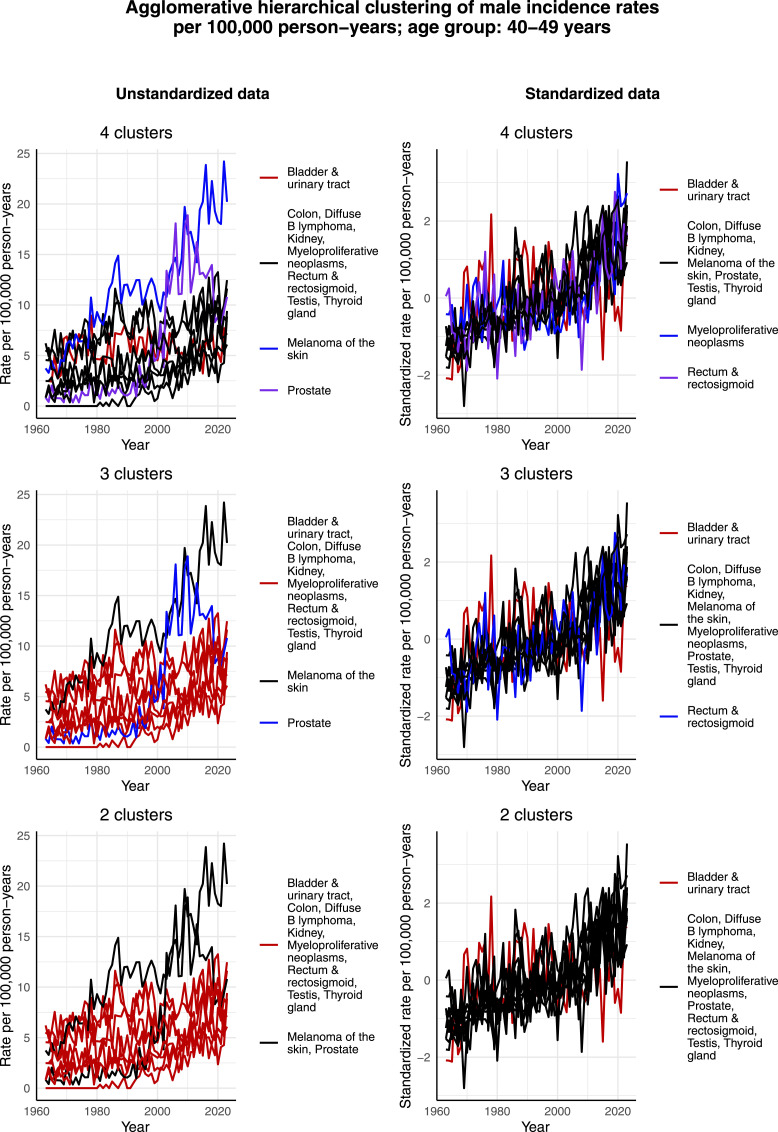
Agglomerative hierarchical clustering applied to the incidence rates per 100,000 person-years of the most common cancers among males aged 40-49 years in Finland

Figure 9 illustrates the clustering results for males aged 40-49 years. Neither in this age group there seems to be large differences in the development of the standardized incidence rates per 100,000 person-years over time between the most common cancers. That is, the majority of these standardized incidence rates per 100,000 person-years exhibit a slightly increasing trend. It is only bladder & urinary tract cancer for which the trend of standardized incidence rate per 100,000 person-years appears, by Figure 9, to be slightly decreasing from the 1990s onward. However, if we compare the years 1990 and 2023 by calculating the percentage change, the standardized incidence rate has actually increased by 26%. This seems to be an artifact of large random fluctuations in the data as we observe, from Figure 9, that the overall trend is still decreasing. Indeed, if we compare the years 1990 and 2021, the rate has decreased by 177%. The maximum yearly increase between years 1990 and 2023 is 750% and the maximum yearly decrease is 500%. These large fluctuations most likely result from small incidences, and it will be interesting to see to which direction the trend evolves in the upcoming years. In terms of the unstandardized data, it is interesting that the incidence rate per 100,000 person-years of testicular cancer appears to be lower than among males aged 30-39 years and consequently does not form its own cluster. Another interesting observation is that in the case of 3 and 4 clusters, melanoma of the skin and prostate cancer both form their own clusters, but in the case of 2 clusters, these 2 cancers are assigned to the same cluster. The reason for this might be the increased incidence rate per 100,000 person-years of prostate cancer during the 2000s, and especially during the early 2000s the rate matches quite well that of melanoma of the skin. On the other hand, the incidence rates per 100,000 person-years of these 2 cancers have developed in a bit different way compared to each other before the 2000s in this age group.

**Figure 10.**
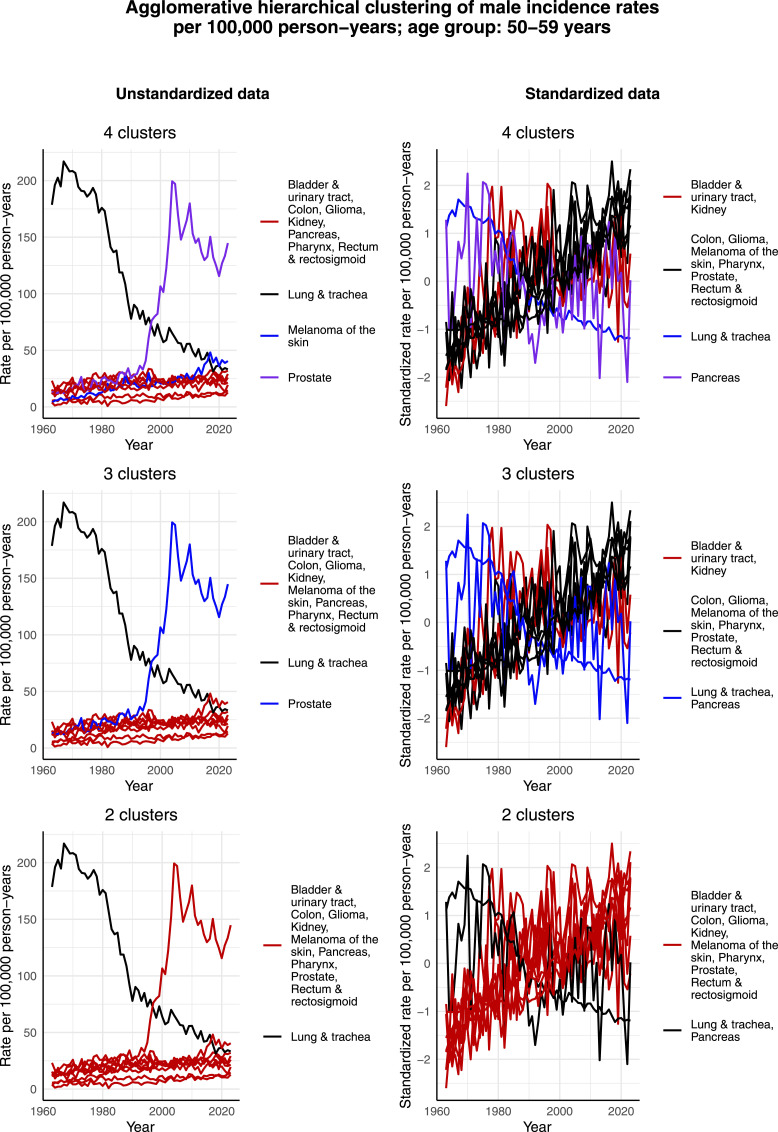
Agglomerative hierarchical clustering applied to the incidence rates per 100,000 person-years of the most common cancers among males aged 50-59 years in Finland

Figure 10 visualizes the clustering results for males aged 50-59 years. In this age group, there is an even higher increase in the incidence rate per 100,000 person-years of prostate cancer, starting from the late 1990s. Consequently, in the case of the unstandardized data and when 3 or 4 clusters are identified, it forms its own cluster. However, in the case of 2 clusters, prostate cancer is assigned to the same cluster as the majority of the most common cancers, excluding only lung & tracheal cancer that in all 3 cases forms its own cluster. The reason appears to be the relatively large decrease in its incidence rate per 100,000 person-years from 1963 to 2023. The decreasing trend of lung & tracheal cancer can also be observed in terms of the standardized incidence rates per 100,000 person-years. Moreover, it appears that also pancreatic cancer exhibits a decreasing trend over time in terms of the standardized incidence rate per 100,000 person-years, and is thus assigned to the same cluster as lung & tracheal cancer in the cases of 3 and 2 clusters.

**Figure 11.**
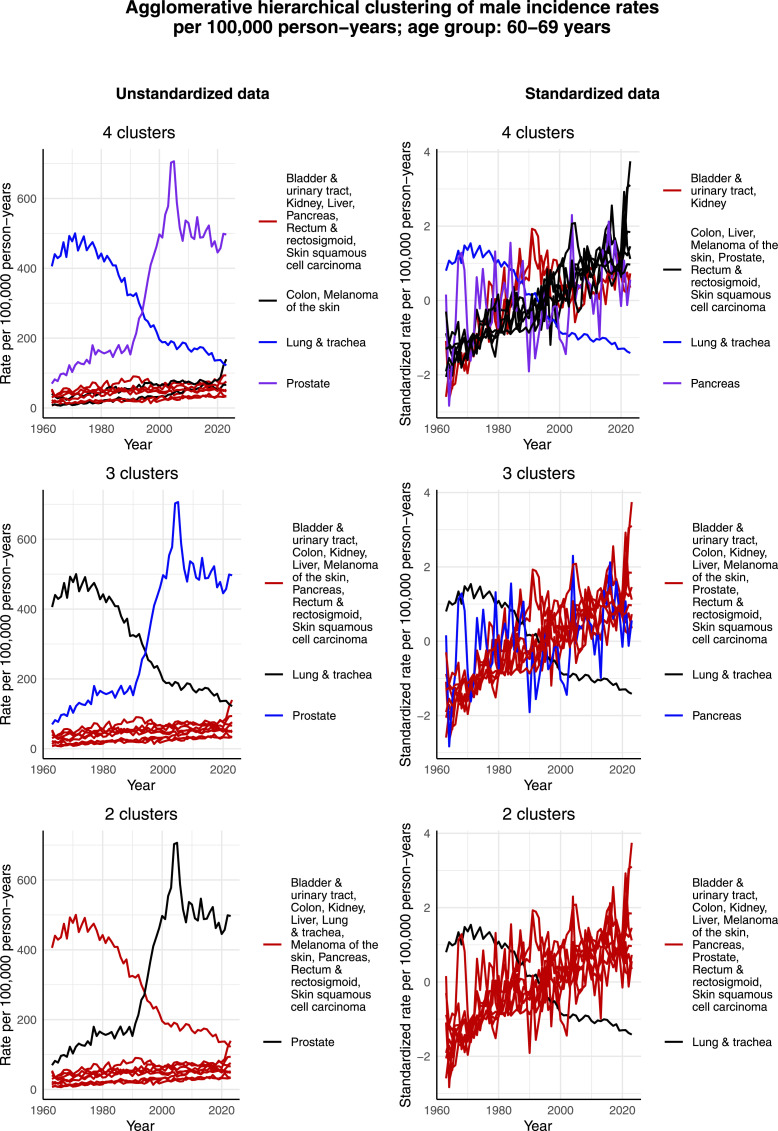
Agglomerative hierarchical clustering applied to the incidence rates per 100,000 person-years of the most common cancers among males aged 60-69 years in Finland

**Figure 12.**
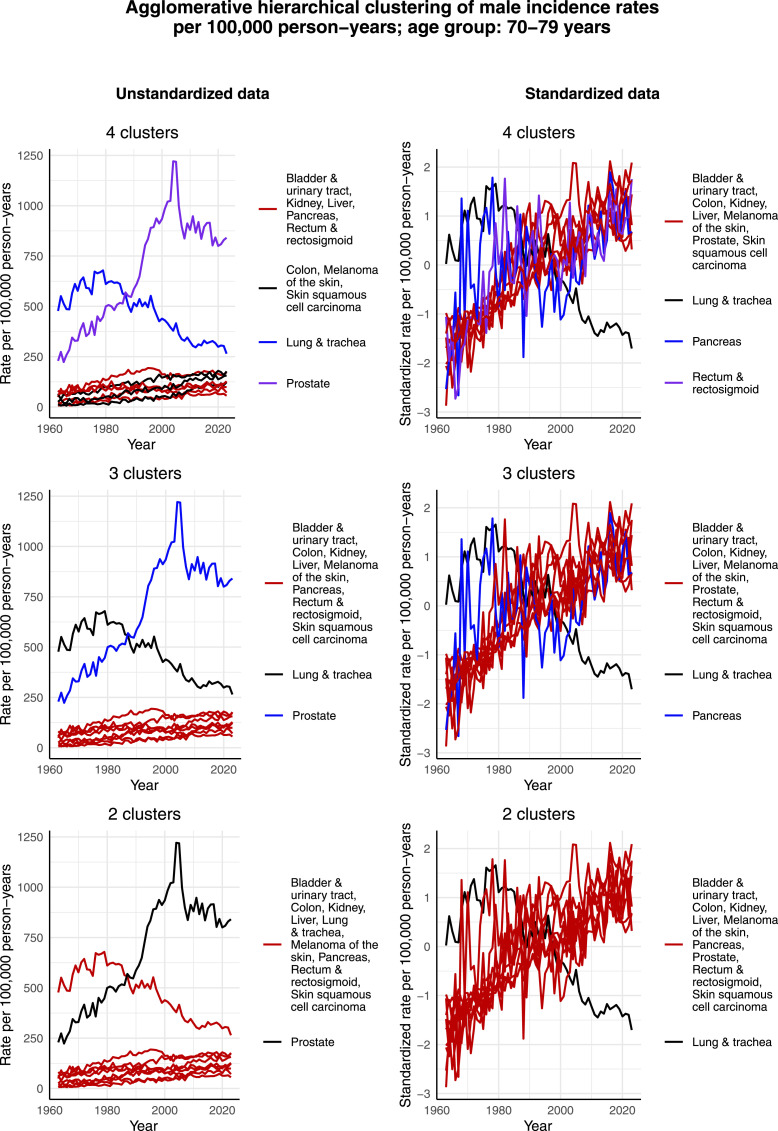
Agglomerative hierarchical clustering applied to the incidence rates per 100,000 person-years of the most common cancers among males aged 70-79 years in Finland

Figures 11 and 12 display the clustering results for males aged 60-69 years and 70-79 years, respectively. The clustering results are relatively similar for these 2 age groups. That is, given the increased incidence rate per 100,000 person-years of prostate cancer during the 2000s, it forms its own cluster in the case of unstandardized data in all cases of 2, 3, and 4 clusters. Although the incidence rate per 100,000 person-years of lung & tracheal cancer has decreased since 1963 in a similar way as among males aged 50-59 years, it forms its own cluster only in the cases of 3 and 4 clusters. It seems that since the scale of the incidence rate per 100,000 person-years of lung & tracheal cancer is closer to the rest of the most common cancers than that of prostate cancer, lung & tracheal cancer is assigned to the large cluster in these age groups, unlike in the age group of 50-59 years. However, in the case of the standardized data in all cases of 2, 3, and 4 clusters, lung & tracheal cancer forms its own cluster. This is reasonable, as its standardized incidence rate per 100,000 person-years has decreased, while the standardized incidence rates per 100,000 person-years of all the other most common cancers appear to have increased over the studied time interval.

## Discussion

When it comes to the research questions of this study, the identified cluster structures were presented above. There are certain characteristics of the cluster structures that can be discussed in more detail. First, in many cases, cancers for which there exists a national screening program, such as breast^19^ and cervical cancer,^26^ or an individualized opportunistic screening tool, such as prostate cancer,^27^ appeared to separate as their own clusters. This is sensible, as screening and testing can affect the incidence rates per 100,000 person-years in a couple of different ways. On 1 hand, systematic screening not only helps diagnosing more cases in general but can also result in the diagnosis of some early-stage tumors that would not have produced any symptoms during the lifetime of the patient, such as in the case of breast cancer.^19^ Research also shows that after the introduction of PSA, or prostate-specific antigen,^28^ testing in Finland, a rapid increase in the incidence of localized prostate cancer can be observed.^27^ On the other hand, since the cervical cancer screening program enables the detection of cancer precursors that are curable,^29^ screening may also have a lowering effect on incidence.

Other cancer types that exhibit a tendency to form their own cluster in multiple cases are melanoma of the skin, in particular in the younger age groups, and lung & tracheal cancer in the older age groups. Research suggests that the increase in the incidence rate of melanoma of the skin in recent decades is largely attributable to increased exposure to UV radiation.^30^ Given this and the fact that people can to some extent control their exposure to UV radiation, the findings of this study may reflect a less consistent adoption of protective measures by people, for 1 reason or another, to take appropriate measures to protect themselves from UV radiation. Lifestyle choices can also have a major impact in the case of lung & tracheal cancer. That is, smoking is the most significant modifiable risk factor for cancer among people who smoke,^31^ and in terms of average relative risk, smokers are 15-30 times more likely to develop lung cancer than non-smokers.^32^ Moreover, gender differences in prevalence of smoking have become smaller over time in Finland. A combination of these 2 factors could explain the emergence of lung & tracheal cancer in its own cluster among females aged 60-69 and 70-79 years. On the other hand, increased awareness of the risks of smoking in Finland could explain the decrease in the incidence rate per 100,000 person-years of lung & tracheal cancer among males.

Yet another interesting observation concerning the identified cluster structures is that even though the incidence rates per 100,000 person-years of prostate and testicular cancer both have increased sharply during the early 2000s, the former can at least partly be explained by the onset of PSA testing, as discussed above, whereas for the latter there is no unequivocal explanation yet.^9^ Nevertheless, it has been detected that the incidence rate per 100,000 person-years of testicular cancer is the highest in the Western countries.^33^ This could suggest that certain environmental or lifestyle factors might contribute to this observed trend.

In terms of the differences and similarities in the cluster structures between different subgroups determined by age and sex, the following can be said. First, due to low incidence rates, random fluctuations in the data appeared to guide the clustering process a bit in the youngest age groups. That is, even after considering the whole population within these youngest age groups in this study, the absolute incidence rates were so low that no clear trends were discovered. In the older age groups, the cluster structures were more meaningful, ie, they seemed to emerge from actual heterogeneity in the data instead of random fluctuations. Additionally, the differences between clusters were bigger in the older age groups than in the younger age groups. Second, large differences in scale, in particular when using unstandardized data, affected the clustering results in all subgroups. Another similarity between all of the subgroups is that standardized incidence rates per 100,000 person-years of the majority of the most common cancers have increased from 1963 to 2023. This could hint at a change in exposure to some risk factors for cancer related to our lifestyle or environment. For instance, research has identified convincing evidence that the risk of multiple cancers, including oesophagus, pancreatic, liver, colon, rectal, postmenopausal breast, endometrium, and kidney cancer, is increased by obesity^31^ which, in turn, has been attributed to the Western lifestyle.^4^ Similarly, physical inactivity has been directly linked to the risk of at least colon, breast, and endometrial cancers.^34^ Moreover, there is convincing evidence that processed meat and alcohol use increase the risk of colon and rectal cancers.^31^ Although these did not emerge particularly in the resulting cluster structures, colon cancer was still identified to be either second or third most common cancer both among females and males aged 60-69 and 70-79 years in Finland in 2023. Nationwide colorectal cancer screening was launched in Finland in 2022. The screening currently applies to people aged 60-70 years, and it will be expanded to include people aged 56-74 years by 2031.^35^ This will possibly affect the incidence of colon cancer in the upcoming years.

## Conclusion

In this study, cluster structures from the Finnish cancer incidence data were identified, and differences, as well as similarities, in the observed cluster structures between different subgroups determined by age and sex were analyzed. Furthermore, a tailored proximity measure was proposed and employed in practice to cluster the cancer incidence data.

Some possible future research directions follow from this study. First, time series representing the development of known substances that cause cancer could be included in the cluster analysis. Second, a similar analysis could be conducted in other countries or worldwide with the aim of obtaining a better understanding of the underlying environmental and lifestyle factors. Third, the cancer mortality data of Finland, which is also available in the Finnish Cancer Registry, could be analyzed using similar methods to uncover cluster structures and trends in it. Fourth, the experiments could be performed with different clustering methods, such as *k*-means. Lastly, from the theoretical point of view, the asymptotic behavior and convergence of the proposed proximity measure could be studied.

## Data Availability

The data used in this study is accessible via the Finnish Cancer Registry cancer statistics application^14^ and has also been included in the Supplemental Material available online.^16^
